# Adaptation of a gender-transformative sexual and reproductive health intervention for adolescent boys in South Africa and Lesotho using intervention mapping

**DOI:** 10.1080/16549716.2021.1927329

**Published:** 2021-06-09

**Authors:** Áine Aventin, Stephan Rabie, Sarah Skeen, Mark Tomlinson, Moroesi Makhetha, Zenele Siqabatiso, Maria Lohan, Mike Clarke, Lynne Lohfeld, Allen Thurston, Jackie Stewart

**Affiliations:** a School of Nursing and Midwifery, Queen’s University Belfast, Belfast, Northern Ireland, UK; b Institute for Life Course Health Research, Stellenbosch University, Cape Town, South Africa; c Department of Psychiatry and Mental Health, HIV Mental Health Research Unit, Cape Town, South Africa; d Dreams Project, World Vision, Federal Way, Lesotho; e Centre for Public Health, Queen’s University Belfast, Belfast, Northern Ireland; f School of Education, Social Sciences, Education and Social Work, Queen’s University Belfast, Belfast, Northern Ireland, UK

**Keywords:** Sexual and reproductive health, adaptation, intervention mapping, adolescent boys, gender transformative

## Abstract

**Background**: Rates of adolescent HIV and unintended pregnancy in southern Africa are amongst the highest in the world. Gender-transformative interventions that address underlying gender inequalities and engage both males and females have been emphasised by the World Health Organisation, amongst others, to target prevention. However, few such gender-transformative interventions have been rigorously developed or evaluated.

**Objective**: To expedite potential impact and reduce development costs, we conducted a needs assessment to inform the co-design, in consultation with local stakeholders, of adapted versions of an existing gender-transformative Relationships and Sexuality Education intervention for use in South Africa and Lesotho.

**Methods**: Adaptation of the intervention was guided by a modified version of Intervention Mapping (IM). This process involved consultation with separate adolescent, community and expert advisory groups and a collaboratively conducted needs assessment, which drew on focus groups with adolescents (8 groups, n = 55) and adults (4 groups, n = 22) in South Africa and Lesotho, and was informed by our systematic review of the literature on the determinants of condom use among adolescents in the region.

**Results**: The findings clarified how the intervention should be adapted, which individual- and environmental-level determinants of condom use to target, and actions for facilitating successful adoption, evaluation and implementation in the new settings.

**Conclusions**: The IM approach allows for a systematic appraisal of whether components and processes of an existing intervention are appropriate for a new target population before costly evaluation studies are conducted. The findings will be of interest to those wishing to rigourously develop and evaluate gender-transformative interventions engaging men to improve health for all.

## Background

### Adolescent sexual and reproductive health in Southern Africa

Unintended pregnancy and HIV/AIDS (human immunodeficiency virus/acquired immunodeficiency syndrome) are major global adolescent health challenges. In 2019, around 170,000 adolescents aged 10–19 years around the world were newly infected with HIV and concerns are that, if current trends continue, hundreds of thousands more will be become HIV-positive in the coming years [1]. Although HIV/AIDS has long been the primary sexual and reproductive health (SRH) issue on the national agenda for South Africa [2], HIV rates are still high: 18% among 15–49 year olds and 6% among adolescents [3]. Further, adolescents consistently present with poorer adherence to treatment and clinical outcomes (i.e. hospitalisation and mortality rates) than adults [4]. Lesotho has the second-highest HIV prevalence in the world, estimated in 2019 at 23% among 15–49 year olds and 10% and 5% among 15–24 year old women and men respectively [5]. Additionally, nearly half (48%) of sexually active adolescents in Lesotho report that they do not regularly use condoms [3].

Adolescent pregnancy is also a pressing health and social issue. Pregnancy is a leading cause of mortality and morbidity for adolescent girls in low- and middle-income countries (LMICs) due to issues including delivery complications and unsafe abortions [6]. It is a significant contributor to gender inequality in education, often resulting in interruption or termination of girls’ schooling, and thus a major impediment to social and economic development [7]. Twenty-seven percent of women in South Africa [8] and 19% in Lesotho [3] are pregnant by the time they are 19 years old. Over half of the 260,000 abortions that take place every year in South Africa are unsafe (i.e. performed by persons lacking necessary skills and/or in an environment that does not conform to minimal medical standards), even though abortion is legal [9]. In Lesotho, abortion is criminalised and unsafe terminations account for up to 50% of inpatient deaths among females aged 13 years and older in some Lesotho hospitals [10].

### Gender transformative interventions for young adolescent boys

The continued existence of high rates of pregnancy and HIV in African adolescents after 30 years of focused attention suggests that there are deeply rooted cultural and socio-economic factors that foster high-risk sexual behaviours. It is now recognised that a key determinant of these global health challenges is gender inequalities plus restrictive masculinities and associated practices that predispose adolescent men and women to the risk of HIV infection and unintended pregnancy (e.g. unprotected/condomless sex, multiple and concurrent sexual partnerships, sex while under the influence of alcohol and/or drugs, and gender-power relations linked to sexual violence) [11]. International health and development frameworks therefore emphasise the importance of working with both boys and girls in order to reduce gender inequality and improve SRH outcomes for all [12,13].

*Gender Transformative* (GT) approaches seek to examine and change harmful gender and power imbalances and encourage men’s role as enablers rather than inhibiters of women’s health and well-being [14–16]. Typically, GT interventions in adolescent SRH seek to address restrictive masculinity norms relating to heterosexual prowess and gendered power relations (e.g. promoting shared sexual decision-making between males and females) and challenge negative gender socialisation (e.g. challenge perceptions that ‘real men’ don’t wear condoms or that girls should not carry condoms) [17,18]. In line with ‘social norms’ approaches, GT interventions generally attempt to harness the misalignment between people’s individual behaviours and attitudes and existing social norms in order to affect change [19]. Strategies often also include changing gender norm attitudes among an influential social group and leveraging their influence as agents of change in their peer groups and communities [20]. Although GT approaches have been applauded for their focus on gender inequalities, evaluations of GT programmes have also been criticised for their lack of methodological scope and rigour [18,21]. Scholars are also beginning to understand that, in order to be truly transformative, GT programmes must also seek to address the intersectional influences of other social factors on gender inequalities including race, ethnicity, sexual orientation, and poverty [14,16,18,22].

While GT interventions recognise that men have a vital yet neglected role in reducing unintended adolescent pregnancy and HIV rates, there is a global dearth of robustly evaluated GT programmes targeting adolescent boys to prevent HIV and early unintended pregnancy. This gap in programming has been noted as a primary objective by a group of global leaders known as the Bellagio Working Group on Gender and Growth [23] as a means of reducing gender inequality by 2030 [24]. Further, a 2019 WHO systematic review of reviews on research engaging men and boys in sexual and reproductive health (SRH) indicated that only 8% of the included reviews (39/462 reviews) reported studies using a GT approach with men and boys, and only 10% of these (4 reviews) focused on adolescent SRH [16]. A second WHO systematic review which examined the components and characteristics of GT interventions involving men and boys determined that only 24% (16/68) of experimental studies used a GT approach with adolescents [25]. Only five of those 16 studies specifically engaged male adolescents in promoting sexual health and well-being (including HIV and STI prevention), desired family size or ensuring the health of pregnant women and girls. Furthermore, only two of those five studies were located in sub-Saharan Africa (1 in South Africa and 1 in Ethiopia). The South African study was a quasi-experimental study that focused on STI/HIV prevention only (not unintended pregnancy). This body of work suggests that the evaluation and implementation of GT interventions in southern Africa is absent and firmly supports the urgent need for conducting and disseminating information about robust evaluation programmes.

### The value of intervention adaptation

In the field of adolescent SRH, scaling-up programmes that have demonstrated impact in a specific context has been recommended by Douglas Kirby as ‘the single most promising strategy for reducing teen pregnancy and STI/HIV’ [26] (p.187). A major challenge with this approach, however, is that the effectiveness of public health interventions is usually linked to the social and cultural context in which they are implemented; thus, interventions that show impact in one context may not necessarily be effective in a new setting or with a new population [27].

Increasingly, therefore, public health researchers are systematically identifying and modifying key intervention components and implementation processes to fit new contexts [28]. This approach may be particularly valuable in LMICs, where resources are limited for developing and evaluating new interventions and where there is a greater reliance on evidence from other settings regarding interventions that show promise in the original context. A recent systematic review of adaptation processes recommends the use of rigourous adaptation frameworks when scaling-up programmes to new settings, although such frameworks are currently used in less than one-third of intervention adaptation studies [28].

Our study aimed to address these gaps in the literature by working with local stakeholders in Lesotho and South Africa (where rates of HIV/AIDS and unintended adolescent pregnancy are among the highest in the world) to co-design contextually relevant adaptations of a SRH intervention guided by a systematic adaptation framework. The gender-transformative *If I Were Jack* (JACK) intervention [29–31], developed for use in the UK, targets adolescent men through a variety of individual, school- and home-based material designed to help adolescents reduce sexual risk-taking (unprotected/condomless sex) and address gender inequalities in SRH. We used a systematic and participatory process, working closely with local stakeholders, to initiate what we believe is the first robust study of a GT adolescent pregnancy- and HIV-focused intervention specifically targeting adolescent men in South Africa and Lesotho, but also applicable and designed to be delivered to adolescent women alongside adolescent men. This paper details the development phase of a planned larger study which will optimise and test the contextually-adapted versions of *JACK* in a feasibility randomised trial.

### Aims & objectives

The aim of the study is to use a systematic and collaborative process to design sociocultural adaptations of the *If I Were Jack* intervention for use in Khayelitsha, South Africa and Maseru, Lesotho.

Study Objectives (repeated in both countries):

- *Convene Project Advisory Groups*: Separate adolescent and community groups were convened in each country. Groups were consulted twice during the project to decide how best to: a) engage with the broader community for a successful project; b) address culturally-sensitive issues; and c) adapt, as needed, the *JACK* educational pedagogies for local contexts.
- *Conduct Needs Assessment*: We conducted an assessment of target population sexual and reproductive health needs and clarified contextually relevant determinants of condom use. This was based on a new systematic literature review and primary qualitative research (focus groups) with local adults and adolescents.
- *Conduct Intervention Mapping Exercise*: Based on findings from the needs assessment (systematic review and focus groups) and in consultation with advisory groups, we conducted an intervention mapping exercise. This aimed to assess the acceptability and feasibility of the *JACK* components for use in these new settings and produce detailed plans for adaptation, implementation and evaluation of the original JACK into new culturally bespoke interventions. These plans formed the basis of proposals for further research funding to develop and evaluate the interventions.

## Methods

### Study design

The research design was grounded in three methodological approaches:

1. The UK MRC (2008) *Framework for the Development and Evaluation of Complex Interventions* to guide the overall research design [32]. This framework helped ensure that this formative work was conceptualised as an MRC ‘Phase 1ʹ development study that would lead to either further development work and feasibility testing at the next stages;
2. *Intervention Mapping* [33] involving evidence synthesis and primary research with local stakeholders to guide the adaptation process. This framework was chosen because it offered guidelines for a systematic process for cultural adaptation; and
3. A participatory approach to research involving consultation with local stakeholders including adolescents, parents, teachers, and community-based service providers as a means of including their unique experiences and needs in relation to matters that affect them [34].

Ensuring methodological rigour was a primary concern and we were guided in this endeavour by Lincoln and Guba’s concept of t*rustworthiness*, incorporating considerations of credibility, dependability, confirmability and transferability [35]. Our approach to incorporating each of these considerations is addressed the relevant sections below.

### Original intervention

*If I Were Jack* [70,83,84] is an evidence-informed, theory-based, gender-transformative (challenges gender inequalities relating to SRH) RSE programme designed to reduce unintended teenage pregnancy and promote positive sexual health. It aims to increase intentions to avoid teenage pregnancy and HIV/STIs by encouraging delayed initiation of sexual intercourse and/or consistent use of contraception and is designed to be delivered in educational settings. It specifically targets boys aged 14–15, however, it can also be delivered to girls and used in same-sex or mixed-class groups. It is designed to promote critical thinking about the social pressures that normally situate teenage pregnancy and its prevention as a female-only issue. Programme components include: an interactive film which tells the story of 16-year-old Jack, who has just found out that his girlfriend Emma is unexpectedly pregnant; classroom materials for teachers containing detailed lesson plans with specific classroom-based and homework activities; a 90-minute training session delivered by RSE specialists to teachers implementing the programme and parent components as described below. The JACK programme and Trial methods are described in full elsewhere [29,30] and more information about the project can found at www.qub.ac.uk/if-i-were-jack.

### Setting

The study was conducted in two countries, Lesotho and South Africa. Lesotho and South Africa differ in terms of culture, language and religion, but they both have very high levels of poverty, unemployment and gender-linked violence [36]. In Lesotho, the study was located in the Maseru district, which consists of the capital city, Maseru (population circa 330,000) and other surrounding peri-urban and rural areas. Lesotho is listed among the ‘least developed countries’ on the Development Assistance Committee (DAC) list of Official Development Assistance (ODA) recipients. Despite huge need in relation to SRH in Lesotho with services mostly centred in the capital, government expenditure on health overall is only around 5% of gross expenditure [37]. In South Africa, the study was located in the peri-urban informal settlement of Khayelitsha (population circa 500,000), which is situated on the outskirts of Cape Town. While South African law guarantees access to SRH services, there remains a large service gap with an economic status gradient evident in places such as Khayelitsha [6].

We chose to work in these countries for a number of reasons. First, they are home to some of the most vulnerable adolescents in Southern Africa who are very much in need of evidence-based SRH interventions. Second, we thought the differences between them (in particular culture, poverty levels, abortion laws and SRH services provision) would allow identification of key factors that can promote or inhibit adoption, implementation and evaluation during future phases of the study. Importantly, the project team in South Africa also had extensive experience working in both country sites.

When compared to the UK, for which the original intervention was designed, South Africa is comparable in terms of reported gender equality indexes, while Lesotho shows stark differences. In 2020, the Global Gender Gap Report [36] ranked the UK 21^st^ out of 153 countries, while South Africa was placed 17^th^ and Lesotho 88^th^. Further, the report highlights that while 29% of women in the UK and 21% of women in South Africa report ever having experienced gender violence, the figure in Lesotho is 62%. Additionally, while unmet need for family planning in the UK stands at 6%, figures in South Africa (15%) and Lesotho (18%) are higher, as are incidences of maternal mortality (7 per 100,00 live births in the UK compared with 119 in South Africa and 544 in Lesotho).

Due to the burden of HIV/AIDS in the region, it is imperative that SRH interventions targeting adolescents incorporate a focus on HIV reduction. For this reason, the adapted intervention will appoint equal focus on the avoidance of HIV/STIs and unintended pregnancy. While we recognise the importance of the full range of contraceptive methods available – the JACK programme encourages the use of condoms alongside another contraceptive method – we focus on condom use because it is the only method of contraception that provides dual protection from STIs and pregnancy. Our definition of ‘unprotected sex’ for this project therefore refers to sex without a condom (‘condomless’ sex). When we refer to ‘contraception’, this includes condoms and all other methods of contraception, unless otherwise specified.

### Project advisory groups (PAGs) and focus group participants

We used a systematic and collaborative process to assess SRH need and plan sociocultural adaptations of the *JACK* intervention for use in South Africa and Lesotho. We employed a participatory approach, working with local stakeholders to obtain contextually relevant information pertaining to the study objectives. This involved the establishment of separate adolescent, community, and expert project advisory groups (PAGs) in each country to consult on how best to: a) engage with the broader community for a successful project; b) address culturally-sensitive issues; and c) adapt, as needed, the *JACK* educational pedagogies for local contexts. Table 1 summarizes the demographic characteristics of the PAGs and study participants. Across both sites, we recruited a combined total of 53 PAG members and 77 adolescent and adult focus group participants. We employed convenience and snowball sampling to recruit focus group participants according to pre-specified inclusion criteria that ensured a mix in terms of gender, age and occupation. The PAGs acted in an advisory capacity only. While their opinions were considered during the IM process, no data were collected from them and their views are not reported in detail in any publication.

**Table 1.** Demographic characteristics of project advisory group members and focus group participants

Project Advisory Group				
Lesotho	Age Range	Gender	Representatives	Geography
Adolescent	13–17	*N* = 9 (5 female; 4 male)	4 Youth NGO; 2 secondary school students; 3 community members	6 urban; 3 peri-urban
Community	18+	*N* = 8 (4 female; 4 male)	2 NGO; 2 health workers; 1 teacher; 1 principal; 1 caregiver; 1 community leader	6 urban; 1 peri-urban; 1 rural
Expert	18+	*N* = 13 (8 female; 5 male)	6 Ministry of Education and Training; 4 NGO; 2 Ministry of Health, 1 UNICEF;	13 urban
South Africa	Age Range	Gender	Representatives	Geography
Adolescent	13–17	*N* = 8 (5 female; 3 male)	8 community members	8 peri-urban
Community	18+	*N* = 7 (5 female; 2 male)	2 social workers; 2 teachers; 2 caregivers; 1 community leader	7 peri-urban
Expert	18+	*N* = 8 (5 female; 3 male)	5 Western Cape Education Department; 2 NGO; 1 Department of Health	13 urban
Focus Group Participants				
Lesotho	Mean Age	Gender	Representatives	Geography
Adolescent	16.1	*N* = 28 (15 female; 13 male)	18 secondary school students; 10 community members	24 urban; 4 peri-urban
Adult	44.2	*N = *12 (6 female, 6 male)	3 caregivers; 2 councillors; 2 pastors, 2 teachers; 1 police officer; 1 village health worker; 1 community leader	12 urban
South Africa	Mean Age	Gender	Representatives	Geography
Adolescent	14.6	*N* = 27 (14 female; 13 male)	27 secondary school students	27 peri-urban
Adult	42.0	*N* = 10 (8 female; 2 male)	2 teachers; 2 caregivers; 2 nurses; 2 community leaders; 1 NGO; 1 social worker	10 peri-urban

### Ethical considerations

The study was approved by the Health Research Ethics Committee of Stellenbosch University [N19/07/081] and the Lesotho Ministry of Health Ethics Committee [ID 215–2019]. Prior to enrolment in the study, informed written consent was obtained from all adult participants. All adolescent participants provided informed written assent and informed written consent was also provided by their caregivers. In order to promote privacy, focus group participants were reminded that they should only share information that they were comfortable sharing publically and asked to verbally agree to ’group rules’ that everything said during the discussion should be considered private. In order to maintain confidentiality, participants were assigned a participant identification number and this number was used for all information collected. No other information that would disclose the participant’s identity was included in transcripts. All focus group participants received R160/M160 in vouchers as well as travel expenses to and from the focus group venue. The research team developed and adhered to a data storage and management plan, which detailed steps for ensuring data quality across researchers and countries and was approved by both ethics committees.

### Data collection and analysis

**Figure 1.**
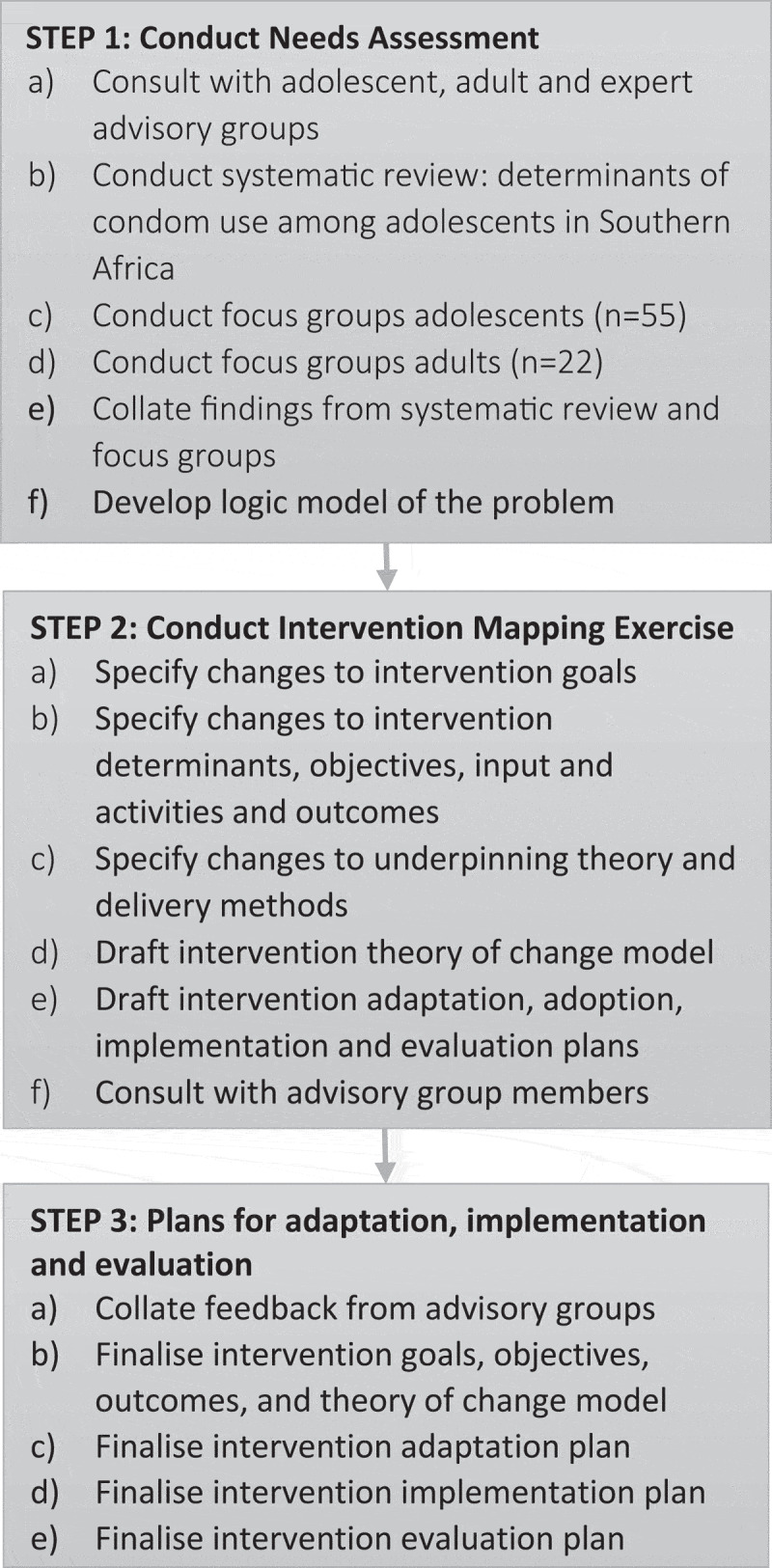
Modified intervention Mapping process

We used a modified version of Bartholomew et al’s [33] Intervention Mapping (IM) approach to frame and inform the data collection and analysis processes. Key components of the IM-informed adaptation process is to conduct a needs assessment (Step 1) and then compare and contrast the components and theory of change from the original intervention with the needs of the new target population (Step 2). Divergences between the existing intervention and the needs of the new population indicate what changes need to be made to the original intervention informing adaptation, implementation and evaluation plans at Step 3. Figure 1 presents the modified version of IM used in this study. We conducted Steps 1 and 2 separately in both countries.

Following initial consultations, and positive feedback from PAGs regarding the potential suitability of adapted versions of the JACK intervention for use in South Africa and Lesotho, we conducted a systematic review of qualitative and quantitative literature on the determinants of condom use among adolescents in Southern Africa, and a series of focus group discussions with the adolescent and adult participants. The aim was to ensure that the adapted intervention would be based on a thorough understanding of the needs of the target population and current community capacity to implement it (Figure 1, Step 1).

The findings of the systematic review, reported elsewhere [38,39] provided information on pertinent population and contextual issues that helped inform the needs assessment process and also informed, in collaboration with PAGs, the development of the semi-structured interview schedule used to collect primary data.

We collected primary data from 12 focus group discussions (6 in each country), eight with 55 adolescents and four with 22 adult participants. Participants represented a convenience sample, recruited via requests for volunteers from non-government organisations on our advisory groups. To help ensure the credibility of the findings (47), the focus groups were facilitated by experienced research assistants who were provided with training to ensure they had adequate knowledge of the subject. Facilitators were also provided with extra training to help them address common challenges (e.g. dealing with embarrassment, ensuring privacy, encouraging the expression of difficult opinions) experienced in conducting focus groups on sensitive topics [40]. Further, the researchers resided in the communities where the study was conducted, and were first language speakers of Sesotho or isiXhosa, the predominant languages in the two sites. The researchers implemented reflexive journals [41] during fieldwork and had regular meetings with the project manager during the process, reflecting on experiences and preliminary findings. Following best practice [35], the focus groups used a flexible format to explore the factors that affect adolescent sexual behaviour and researchers spent time at the end of each session checking and confirming that the key messages they heard were accurate. During the focus groups, the facilitators spent 2–3 hours with the groups, using the pre-designed topic guides to ask about views on knowledge, attitudes, issues around self-efficacy, intentions, perceptions of risk, and perceptions of social norms relating to sex. They also explored the relevance of the *Jack* intervention materials with the participants and elicited suggestions on how to adapt them in the two African study sites. While data saturation was not a guiding feature of the research design, mainly because of time and resource constraints, we are confident that the rich data provided from 12 focus groups, triangulated with systematic review findings is adequate to support the potential transferability of findings [35]. To help ensure dependability of the findings [35], we prepared and adhered to a study protocol containing a detailed description of the methods and established an audit trail of the data collection and analysis processes.

The focus group interview topic guides were developed in English by the local research teams, translated into Sesotho and isiXhosa, and pilot tested with the PAGs. The focus group discussions were conducted in the respective first languages of the participants in both countries. All focus groups were video recorded, transcribed verbatim in the language of the interview, and translated from Sesotho and isiXhosa into English.

Two authors (SR, ÁA) submitted the transcripts to thematic analysis [42], coding the data to help identify patterns in the data and recurring themes and meeting regularly to discuss and confirm coding accuracy, thereby helping to ensure confirmability of the findings [35]. The analysis process followed Braun and Clarke’s [43] six steps. First, we familiarised ourselves with the data by reading the transcripts twice. Second, the transcripts were imported into ATLAS.ti 8 [44] to conduct initial coding. This involved coding sections of data using open theoretical coding linked to our overall research objectives of understanding the determinants of condom use and perceived intervention needs of the participants. Examples of the codes applied at this stage are provided in Table 2. At step three we examined the codes to see if they fitted clearly into a theme, for example, several codes related to how boys and girls were treated differently and these were combined into an initial theme of ‘gender inequalities’. At step four we reviewed the initial themes in relation to the overall data set and to see if they made sense. We followed the guidance of Braun and Clarke [43] in relation to asking questions about whether the data fits the theme, if the themes overlap, if there are sub-themes etc. For example, we considered the theme ‘lack of diversion for adolescents’ in detail and decided that there was enough data and differences for it to constitute a separate theme rather than sub-theme of barriers to condom/contraceptive use. Finally, before the sixth step of writing up the findings, we followed Braun and Clarke’s instructions to ‘define the themes’ or describe what they meant ‘in essence’. This involved, writing reflexive memos [41] while reviewing and coding the transcripts to help synthesise emerging themes across the research domains and paid particular attention to interrogating the data to test underlying assumptions to update the intervention’s theory of change model . In practice this reflexive writing about codes and categories was a part of the entire analysis process, often involving reflection on what was happening in a particular discussion or statement and comparing with other similar or different discussions or statements. The memos were often also used for the researchers to reflect on and challenge their own interpretations of the findings [41].

**Table 2.** Codes, themes and sub-themes from focus group discussions

Focus Group Themes and sub-themes	Illustrative Participant Quotes
Theme 1: Unequal gender norms promote sex without a condom	
Codes: - Boys carry condoms, girls do not - Boys make sexual decisions - Adolescent pregnancy is stigmatised for girls and a status symbol for boys -Boys do not like using condoms because they reduce pleasure	*- ‘boys are very naughty, we have that rulership, we tell ourselves that I’m going to rule this girl’* (adolescent, male, South Africa) *- ‘so if the man feels like it is time, the woman doesn’t have a say; so they just do it; get pregnant and the rest is history’* (adolescent, female, Lesotho) *- ‘it is always the case that boys [rather than girls] take protection’* (adolescent, female, Lesotho)
Sub-theme: Transactional sex – material needs and power	
Codes: - Some girls have sex for money to make ends meet - Some girls engage in transactional sex for material goods from older men ‘blessers’ - Girls are unable to request condom use in transactional relationships - Unequal power relations	- *‘due to poverty in their homes, they need to make a living out of prostitution’* (adolescent, female, Lesotho) *- ‘the blesser does not want to use a condom and because she wants the money and she knows that she will get the money, she will then allow that’*. (adult, female, South Africa)
Theme 2: Peer Influences on sexual behaviour	
Codes: -Perceived norms (everyone is having sex) -Peer pressure (better having sex without condom) - Early sexual experience seen as a sign of maturity and masculinity for boys - Having multiple partners is a status symbol for boys	- *‘one friend will be telling the other about sex, and they will be curious to feel how it is’*. (adolescent, female, Lesotho) *- ‘for us boys when we talk, you will hear them saying: “if you can sleep with that girl, you are a top dog” and they get respect from that’* (adolescent, male, Lesotho) *- ‘it’s status when they talk, when someone is telling the other one that I have this number of girls’* (adult, female, South Africa)
Sub-theme: Substance use leads to unprotected/condomless sex	
Codes: - Drinking and taverns are a common part of life - Substance use as a reason for unprotected/condomless sex - Sexual assault when under the influence of substances	- ‘*there is no one that does not go [to] the tavern, there is no one that stops the children to not go around at night’* (adult, male, South Africa) - *‘when [a] young person uses alcohol, they may engage in activities that could lead to unplanned pregnancy’* (adolescent, female, Lesotho) *- ‘even if she does not want to but because she is drunk she will not have the strength to fight him off, and end up doing it’* (adolescent, male, South Africa)
Theme 3: Access and Barriers to Condoms and Other Contraception	
This theme consisted of three sub-themes: 1) Stigma in the community; 2) adults talking about sex is culturally taboo; and 3) Misconceptions about condoms and other contraception	
Sub-theme: Stigma in the community around adolescent sexuality	
Codes: -Adolescents know where to access (knowledge and availability not a barrier) - Stigma as a barrier to access - Fear of clinic staff - Fear of being caught carrying condoms	*- ‘imagine getting in here going to ntate [sir] there to ask for condoms, he is going to ask me so many questions’*. (adolescent, male, Lesotho) *- ‘They are afraid to be looked in a bad manner because the nurses sometimes have no patience, they talk in a bad way’* (adolescent, female, Lesotho)
Sub-theme: Adults talking about sex is taboo	
Codes: - Adult-child talking about sex is taboo, sinful, perverted - Talking about sex promotes sex - Parents do not have SRH knowledge themselves - Teachers uncomfortable teaching relationships and sexuality education	*‘it goes back in the day when for us Basotho, it was a taboo to sit your child down and talk to them about sex*’ (adult, female, Lesotho) *‘it is like a sin to introduce a sex topic to the child’* (adult, male, Lesotho) *‘if you tell a child that there is something called sex, they will feel eager and want to try it’* (adult, female, Lesotho) *‘it is still hard for us to talk to our children about sex because it was never done to us’* (adult, female, South Africa) *‘At school, there is a teacher that teaches Life Orientation. He skips the part about reproduction all the time’* (adult, female, South Africa
Sub-theme: Misconceptions about condoms and other contraception	
Codes: - Condoms waste time - Condoms are a ‘bad omen’ (bad luck for current relationships and the future fertility) - Condoms reduce male pleasure - Female contraception causes infertility	*-‘[Condoms] are a bad omen, and they delay [men] to do their stuff [referring to sexual climax]’* (adolescent, male, Lesotho) *- ‘there is no way you can eat a lollipop with is cover’* (adolescent, male, Lesotho) *- ‘some are hearing other children’s opinions about contraceptives maybe say it is not right one day when you are married you might not get children in future’* (adolescent, female, South Africa)
Theme 4: Lack of diversions for adolescents – activities for idle hands	
Codes: - Idle adolescents - Not enough programmes - Adults reported there was nothing else exciting for young people to do (e.g. community, after-school programmes etc.) and that was part of the reason why they engaged in substance misuse and unprotected/condomless sex	*- ‘there should be something that keeps one busy, because if someone idles, they get to have a lot [of bad] thoughts’* (adult, male, Lesotho) *- ‘there must be a programme that will look after children after school so that they do not walk around in the streets, so that the streets can be quiet while the children are in that programme’* (adult, female, South Africa)
Theme 5: Responses to the JACK intervention	
Codes: - Opportunities for discussion and self-reflection welcomed - Interactive modality and story-line of film acceptable - Adolescents did not want to complete the homework exercise with parents/caregivers - All other activities acceptable with minor contextual modifications - Parent/caregiver animated films acceptable - Delivery by older peers rather than teachers -Mixed views about whether should be delivered in schools or community settings - Potential parental resistance to JACK in Lesotho	- ‘*I* [am] *able to tell other people about new things that I learned, maybe they also did not know about it and they will get to know it and have more knowledge’* (adolescent, male, Lesotho) *- ‘is better because they [characters in the IVD] discuss the issue, unlike here, here we do not discuss, when there is something bothering you, you keep it to yourself’* (adolescent, female, South Africa) *- ‘where those questions pop up, I am able to empathise with the characters as to what I would do while in a similar situation’* (adolescent, female, Lesotho) *- ‘it is not easy for me to watch it [the If I Were Jack IVD] with an adult’*. (adolescent, male, Lesotho) *- ‘it is nice, I liked it, things that are written here, I liked most of what is on this paper’* (adolescent, female, South Africa) -“if it is ‘*delivered by someone older, there might be some resistance and it might bore them; but if it is delivered by their peers, it is easy for the message to cut across*’ (adult, male, South Africa) - “*I think if parents are involved, it would be just to teach them how to talk to their child, and when things like this one have happened, they should be able to address them together with their child.’* (adult, female, Lesotho) - “*Whenever something new comes up* [in relation to sex and HIV], *it is not often that it gets a good reception [from parents]. All you need to know is that there will be some resistance at first, but that doesn’t say if they refuse; you will have failed*. (adult, female, Lesotho)

Transcripts from each country were analysed separately and then combined in the final synthesis. Any similarities and differences between the countries were highlighted. Most of the resulting themes presented below are at the descriptive rather than abstract level. We agreed the presentation of semantic (rather than latent) level themes was in line with our aim to identify the explicit expressed needs of participants and would facilitate the synthesis of findings across the different data sources.

As illustrated in Figure 1 (step 2), we used the findings from the needs assessment to conduct an IM exercise led by ÁA in consultation with the wider team and PAGs. This process relied heavily on data source triangulation (systematic review and focus group findings). The synthesis process began with a consideration of the findings from the needs assessment (review and focus group findings) and consultation with PAGs, and a whole-team discussion of these based on the questions below.

1. Why do young people in South Africa and Lesotho have unprotected/condomless sex (what are the determinants of unprotected sex)?
2. Which of these determinants can we target with the adapted intervention?
3. What components of the JACK intervention are acceptable for use with adolescents in South Africa and Lesotho and which components are not acceptable?
4. Are additional components needed?
5. What applications (activities) are acceptable for use with adolescents in South Africa and Lesotho and which components are not acceptable?
6. What additional applications (activities) are needed?
7. Are there any implications for adoption of the programme among the new target population?
8. Is the JACK implementation process feasible in South Africa and Lesotho? If not, what alternative implementation processes need to be applied/tested?
9. Is the JACK evaluation process feasible in South Africa and Lesotho? If not, what alternative evaluation methods need to be applied/tested?

The answers to these questions were used to update the project’s original logic model of the problem; developing matrices that specified changes to intervention goals, objectives, outcomes, underpinning theory and delivery methods; and producing a draft ‘theory of change model’ and draft adaptation, adoption, implementation and evaluation plans. These were finalized following consultation with advisory group members.

## Results

### Step 1: needs assessment

Table 2 presents an overview of the themes and sub-themes, with exemplary quotes, relating to the determinants of condom use that emerged from focus group discussions. Four overarching themes (unequal gender norms, peer influences, access and barriers to condoms and other contraception, and a lack of diversions for adolescents), including five sub-themes, related to the perceived determinants of contraceptive/condom use among adolescents in South Africa and Lesotho. A fifth overarching theme related to responses to the JACK intervention.

The first theme related to reports of *unequal gender norms* that place sexual decision-making, sexual pleasure and condom use as male privileges and prevent females from carrying and using condoms without stigma. A sub-theme related to unequal gender norms, *transactional sex*, also emerged as important, with participants reporting that some females engage in condomless sex with older males in order to ‘make ends meet’ or receive money or material goods.

A second theme related to *peer influences*, in which peer pressure, restrictive masculinities and misalignment between perceived social norms and participant attitudes were evident. Participants talked about *others*, specifically other males, engaging in sex without a condom, early sexual initiation or multiple partnerships because it increased their sense of status. Participants were also under the impression that most people of their age were having sex. A sub-theme relating to peer influences, *substance use*, highlighted perceptions among both adolescents and adults that unsupervised alcohol and drug use in local ‘taverns’ was a key determinant of unprotected/condomless sex. Participants also reported that sexual assault was common under the influence of alcohol and drugs.

A third theme *access and barriers to condoms and other contraception* was composed of three sub-themes: *stigma in the community, adults talking about sex is culturally taboo, and misconceptions about condoms and other contraception*. Central were reports that SRH services were stigmatising of adolescent sexuality and contraceptive use and not ‘youth friendly’, and adolescent fears about speaking with adults, including professionals and caregivers, about sex and contraception. Participants agreed that adults speaking with young people about sex was culturally taboo in both countries and such conversations were sometimes seen by adults as ‘perverted’ or ‘sinful’. This was also evident among teachers who felt uncomfortable teaching about SRH and judgemental attitudes expressed by clinic staff when young people sought contraception. While adolescents reported knowing *where* to access contraception, it appeared that the possible social repercussions of doing so often acted as a barrier to using the information. A third barrier related to negative views about contraception. These included beliefs that contraception causes infertility and that condoms are a ‘bad omen’ and reduce male pleasure.

A fourth theme relating to the determinants of contraceptive use among adolescents related to *a lack of diversions for adolescents*. Adults reported there was nothing else exciting for young people to do in their communities and that was part of the reason why they engaged in substance misuse and unprotected/condomless sex.

A fifth theme related to *responses to the JACK intervention*. Overall, participants were overwhelmingly positive about the intervention materials, mostly suggesting only minor modifications to make the materials more culturally appropriate. Reflecting the social taboo surrounding adult-child conversations about sex, a key concern among adolescent participants was that they not be asked to engage in the parent-child homework exercise with their parents/caregivers. While they were supportive of an educational component for parents, most said they would not feel comfortable engaging with them on the subject. Equally, both adolescent and adult participants felt that the intervention should be delivered by ‘older peers’ rather than teachers and while there were mixed views about whether the intervention should be delivered in schools or community settings, there was agreement that both would likely be optimal. Participants in Lesotho indicated that it was likely that there would be some resistance to the programme by parents/caregivers. They suggested that framing the intervention as ‘sexual and reproductive health’ related rather than ‘relationships and sexuality’ focused would help increase acceptability.

These findings were triangulated with results from the systematic review (reported in full elsewhere) [38,39]. In summary, the review synthesis supported findings from the focus groups and also highlighted a number of environmental-level barriers and facilitators not mentioned by focus group participants. Key themes presented in the review included: pervasive unequal gendered norms; social norms reflecting negative constructions of adolescent sexuality and contemporary family planning; economic, political and community level barriers including lack of policy support for condom use, poverty, and lack of youth friendly SRH services and comprehensive sex education; adverse interpersonal influences involving sexual partners, peers and parents/caregivers; and negative attitudes and beliefs about condoms and condom use among adolescents.

**Figure 2.**
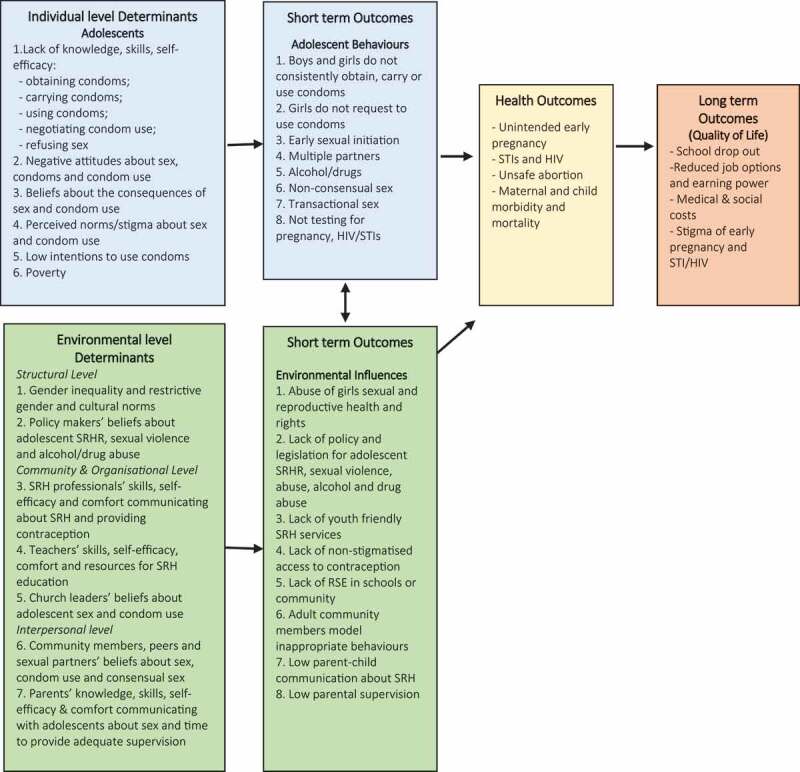
Logic model of the problem

Findings from the needs assessment were used to develop the ‘logic model of the problem’ illustrated in Figure 2. This model illustrates the adolescent (individual) and environmental level determinants of contraception and condom use that emerged from the needs assessment and the reported and theorised negative individual behaviours and environmental influences that result. Individual behaviours and environmental influences are recognised as mutually influential and can lead to negative short/medium term outcomes including unintended pregnancy and HIV/STIs and longer-term quality of life impacts including school-drop out, reduced job options and earning power.

### Steps 2 & 3: intervention mapping exercise and adaptation plan

The needs assessment findings and ‘logic model of the problem’ presented in Figure 2, informed development of the intervention adaptation plan. In order to populate the adaptation plan, the needs assessment findings were considered in relation to their implications for adapting the original intervention goals; targeted individual and environmental level determinants; intervention inputs, activities and outputs; proposed behavioural outcomes, theoretical foundations and delivery methods and detailed adaption activities were noted and applied. While most of the adaptations involved minor adjustments to the intervention materials to optimise cultural appropriateness, the needs assessment indicated a number of findings that highlighted key differences between the new and original populations and contexts. Specifically, these related to the apparent magnitude of influence on adolescent condom use of unequal gender norms and gendered power relations relating to SRH; negative peer influences promoting restrictive masculinities and substance use; adolescent misconceptions about condoms; and a number of environmental-level influences that acted as barriers to condom use including cultural taboo surrounding adult-child conversations about SRH with caregivers, teachers and health professionals. Evident also were key environmental-level influences including a lack of policy and legislation protecting and promoting adolescent SRH, a lack of youth friendly SRH services, a lack of comprehensive SRH education in schools, and the influence of poverty on young women’s engagement in transactional sexual relationships with older men. The adaptations that result from these findings are summarised in Table 3.

**Table 3.** Intervention adaptation plan

ORIGINAL INTERVENTION	PLANNED ADAPTATIONS
INTERVENTION GOALS	
‘To reduce unintended pregnancy and promote sexual health and wellbeing’	Change goals to directly highlight aims to reduce HIV/STI rates and gender inequalities in SRH, as well as unintended pregnancy. ‘To reduce unintended early pregnancy, HIV and STI rates and gender inequalities in sexual and reproductive health’
INDIVIDUAL LEVEL DETERMINANTS TARGETED BY THE INTERVENTION	
FOR ADOLESCENTS: Knowledge Skills Beliefs about consequences Self-efficacy Gender role norms Normative beliefs	Add: ‘Beliefs about barriers to condom use and contraception’
ENVIRONMENTAL LEVEL DETERMINANTS TARGETED BY THE INTERVENTION	
FOR PARENTS/CAREGIVERS AND EDUCATORS: Knowledge Skills Self-efficacy	Add: ‘Beliefs about speaking to young people about SRH’
INTERVENTION INPUTS AND ACTIVITIES	
FOR ADOLESCENTS: Interactive video drama (IVD) Group activities Homework activity FOR PARENTS/CAREGIVERS: Animated films Parent/Caregiver factsheet FOR EDUCATORS: Training Materials	Amendments: IVD uses local actors and is filmed in local areas. All activities and materials are contextually appropriate (e.g. mention local statistics and services) Activities increase current focus on gender inequalities, consensual sex and substance use An activity that focuses on transactional sex is added Activities are amended to mention appropriate local barriers to condom/contraception use and misconceptions about condoms and other contraceptives Lesotho materials are amended to account for the criminalisation of abortion An activity focused on the dangers of unsafe abortion is added for both countries An activity targeting LGBTQI adolescents is added Homework activity is replaced with an individual activity that helps young people to safely identify an older trusted adult that they could speak to about SRH Parent/caregiver activities are amended to address local concerns, particularly addressing cultural taboo relating to adults speaking to children about SRH and increasing knowledge that talking about SRH does not encourage sex Parent/caregiver/educator materials refer to SRH education rather than relationships and sexuality education All online activities are coupled with paper versions for those with no internet access All activities should refer to SRH rather than ‘sex’ or sexuality as this is more taboo
PERFORMANCE OBJECTIVES (INTERVENTION OUTPUTS)	
FOR ADOLESCENTS: Knowledge: Know how to prevent Unintended Early Prgnancy (UEP)/HIV/STIs; discounting myths about condoms; know it is good to wait until ready to have sex; know where to access contraception/safe abortion and find sexual health information and support; know that condoms prevent UEP/HIV/STIs. Skills: communicating with peers, partners and adults (health professionals, teachers); using condoms and other methods of contraception Beliefs about consequences: Believing that when a person is ready and prepared, relationships, sex and pregnancy can be positive experiences; believing that UEP/HIV could be a challenging experience which would impact on current life and future goals and plans; believing in the need to take action in order to avoid negative consequences Self-efficacy: Communicating with peers, partners and adults (parents, educators, health professionals); obtaining condoms/contraception; negotiating condom use; using condoms/contraception correctly; in SA obtaining safe abortion. Feeling confident about being able to communicate about sex and UEP, say no to sex, and obtain and use contraception Normative beliefs: Believing that peers are not having sex; Believing that peers always use contraception when they have sex; Believing that peers should not pressure others to have sex. Gender role norms: believing that both men and women have roles and responsibilities in avoiding UEP/HIV; both men and women should carry condoms; sexual pleasure is not a human right; consensual sexual relationships; ‘real’ men respect women’s sexual decisions; planning for and expecting positive sexual experiences.	Add: Knowledge: know what is consensual and non-consensual sex; know about the possible negative consequences of transactional sex; know how to evaluate relationships; know where to seek help for unhealthy or abusive relationships. For Lesotho, remove ‘know where to access abortion’. For both countries; know about the risks of unsafe abortion and know about post-abortive care. Skills: communicate expectations with sexual partners; avoiding or leaving unhealthy relationships Beliefs about consequences: believing that unhealthy relationships will impact negatively on current life and future goals Believing that gender equal relationship can be happy and fulfilling Self-efficacy: Confidence in ability to communicate personal expectations, preferences and limits Normative beliefs: Add – Believing that peers are not having sex until they feel ready; believing that peers always use condoms when they have sex, even in monogamous/steady relationships; believing that peers should not pressure others to not use condoms, believing that sexual partners should not pressure each other not to use condoms; believing that condom use is not a sign of a distrust in relationships. Gender role norms: Add – Believing that women have the right to make decisions about sex; believing that women have the right to refuse sex; believing that women have the right to request the use of condoms and to refuse sex if condoms are not available; believing that women in transactional relationships have the right to refuse sex without condoms; believing that using condoms does not emasculate men.
BEHAVIOURAL OUTCOMES	
Delayed age of sexual initiation Avoidance of unprotected/condomless sex Condom use Contraceptive use Gender equality	Add: Safe abortion/post-abortive care Healthy relationships Testing for HIV/STIs/pregnancy Asserting gender equal relationships
THEORETICAL FOUNDATIONS	
Theory of Planned Behaviour Transtheoretical Model of Behaviour Change Social Cognitive Theory Social norms Theory Socio-cultural influences (gender, religion, social class) Gender-transformative theory	Consider/Add: Theories of stigma Theories of power
DELIVERY METHODS	
Delivered by trained teachers Delivered in Post-primary schools	Change: Delivery by trained young adult facilitators Deliver in schools and community settings Consider/Add: Drama narration versions of films delivered via local radio

**Figure 3.**
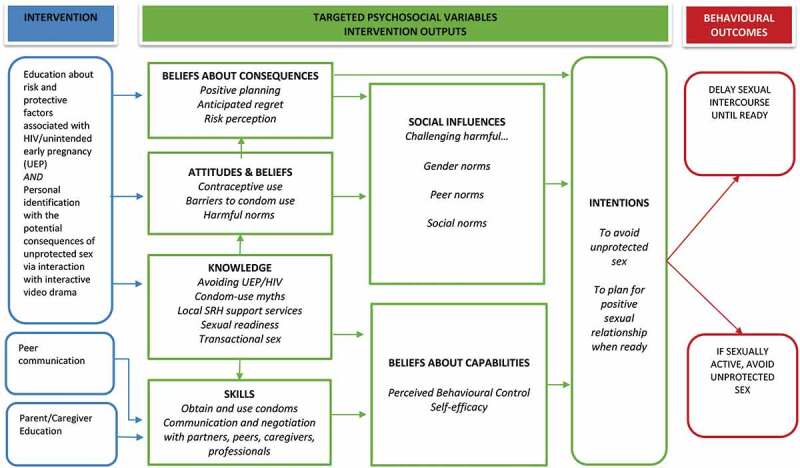
Emerging theory of change model

The final stage in the IM process resulted in an emerging ‘theory of change model’ (see Figure 3) illustrating the adapted intervention theory of change as well as plans for evaluation of the intervention. Findings from the needs assessment plus feedback from the PAGs indicated that a revised version of *JACK* would be acceptable in both Lesotho and South Africa. However, we also heard concerns about the acceptability of the intervention to parents in Lesotho and there were mixed views about whether it was most appropriate to deliver the intervention in schools and/or community settings. As a result, the decision was made to include both an intervention optimisation phase and feasibility trial in both school and community settings in both countries. The study will include an optimisation phase during which intervention materials, including versions of the interactive video drama for each country, will be developed and piloted with adolescents and caregivers in schools and community settings. This will be followed by feasibility randomised trials in both countries.

## Discussion

Evidence-based SRH interventions that engage adolescent boys as well as girls can help transform unequal gender norms that underlie high rates of HIV/AIDS and unintended pregnancy in LMICs. Although high-fidelity implementation of existing interventions is one of the most promising avenues for impact [26], social and cultural differences between countries and populations mean that successful programmes often need to be adapted before being used in new settings. Identifying appropriate adaptations requires rigourous formative work prior to evaluating scaled-up interventions. While adaptation necessarily requires investment of time and resources, it is increasingly recognised that, in the longer term, this may be more efficient and ethical because it will help increase acceptability of the intervention and avoid potential implementation failure because intervention materials and/or delivery methods do not fit the new sociocultural setting [27,45].

Using an adapted version of Bartholemew et al’s [33] IM to structure the process, local stakeholders worked with us to interpret the findings of a needs assessment based on a systematic review of the literature and primary research with adolescents and adults in South Africa and Lesotho. This enabled us to target the SRH intervention to fit the needs of the two study populations and clarified contextually relevant determinants of sexual risk-taking. Within a relatively short time period of eight months, this formative work helped us to produce detailed plans for adaptation of *JACK* into new culturally bespoke interventions as well as locally-developed theory of change models, evaluation and implementation plans. These clarified: how *JACK* should be changed for use with the new populations; which individual- and environmental-level determinants to target; and actions for facilitating successful adoption, evaluation and implementation in the new settings. These plans will directly inform intervention optimisation and feasibility testing during the next stages of the project.

Important differences between the UK and new contexts were revealed. Key findings included insight into the magnitude and influence of pervasive unequal gender norms in southern Africa as central barriers to condom use among adolescents. Reflecting findings by others [46,47], this suggests that GT-focused SRH interventions are much needed in this context. The needs assessment indicated an acute awareness of the misalignment between participant attitudes in relation to gender equality (i.e. that existing gender socialisation and gendered power relations unfairly favour males) and the realities of gender inequalities in SRH as experienced by adolescents (i.e. that unequal gender norms continue to restrict both males and females). There was also strong agreement that gendered stigma related to adolescent sexual behaviour and contraceptive use as well as gendered power dynamics in transactional relationships need to be urgently addressed in both countries. This suggests that while the original intervention theory of change was relevant for this new population and context, there was a need to consider more closely how the intervention might help young people challenge stigma and obtain equality in sexual decision-making, even in transactional relationships. Central to this is consideration of theories of power and stigma [48–50] and the literature on how gender attitudes are shaped in early adolescence [22,51] when adapting intervention activities for these new contexts.

The findings also suggest that other social influences, including peer norms, substance use norms, and taboo associated with adult-child conversations are key determinants of contraceptive use in southern Africa. Again these support previous research [46,47,52,53] and indicate that while the original intervention theory of change and intervention materials are appropriate for use in these contexts, adaptations to intervention materials will need to specifically address local peer norms and safe sexual behaviour in the context of substance use, and employ the recommended peer facilitators to deliver the intervention. Equally adaptations need to be cognisant of the need to engage parents/caregivers and educators while also being sensitive to the existence of cultural norms that might act as barriers. The continued involvement of parent/caregiver and educator stakeholders during the intervention development and optimisation stage will be central to this [54].

The findings also revealed important differences between the two African countries, not least of all the desire of participants in both countries for programme components, including the interactive video drama, to present characters in recognisable locations in their own countries speaking their own language and referring to accurate country-specific services and issues. Another key difference between the countries was the proposed implementation of the intervention in schools only in South Africa but in schools and community groups in Lesotho. Further, while participants in South Africa felt that parents/caregivers would simply not engage with the intervention, participants in Lesotho warned us to expect some backlash from parents/caregivers, whom they anticipated would feel strongly about the inappropriateness of teaching adolescents about sexuality. These findings highlight the value of taking the time to systematically identify and modify key intervention components and implementation processes to fit new contexts [27,28].

### Strengths and limitations

The strengths of this study lie in its use of a systematic process, involving key local stakeholders and a multidisciplinary international team, to conduct a needs assessment that provided contextually rich data for informing the adaptation of an evidence-based intervention. Equally, the study employed robust, rigourous methods to help ensure trustworthiness and transferability of findings. While the primary data emerged from a relatively small sample of focus group participants, thereby limiting external validity, we believe that the triangulation of findings across data sources including a systematic review of literature relating to condom use among adolescents in southern Africa, alongside transparent reporting of methods used increase the value of the findings to other researchers and their relevance and transferability to other countries in southern Africa.

## Conclusion

This study used a systematic process to co-design culturally appropriate adaptations of an evidence-based gender transformative SRH intervention for adolescents in South Africa and Lesotho. It addresses evidence gaps relating to a lack of robustly evaluated GT sexual and reproductive health interventions for adolescents and reports a replicable adaptation process guided by a systematic framework. The IM approach provided a systematic framework for intervention adaptation and helped encourage meaningful participation of stakeholders. The process was facilitated by the combined skills and knowledge of an international multidisciplinary planning and development team comprising academics and practitioners, as well as end-users. It provides the foundations for future research to develop and evaluate a GT intervention with the aim of reducing the rate of HIV and unintended pregnancy among adolescents in southern Africa.

## Data Availability

Data are stored at Queen’s University Belfast. Because the data consists of interviews with sensitive personal information, data will not be shared.

## Paper context

This study addresses global health evidence gaps relating to a lack of robustly evaluated gender-transformative sexual and reproductive health interventions for adolescents. Further, it provides an exemplar of a replicable adaptation process guided by a systematic framework that could be applied in diverse fields of global public health.
